# Sternal Wound Reconstruction Following Deep Sternal Wound Infection: Past, Present and Future: A Literature Review

**DOI:** 10.3390/jcdd11110361

**Published:** 2024-11-07

**Authors:** Arwa Khashkhusha, Sundas Butt, Mariam Abdelghaffar, William Wang, Asveny Rajananthanan, Sakshi Roy, Bakht Noor Khurshid, Mohamed Zeinah, Amer Harky

**Affiliations:** 1 School of Medicine, Faculty of Health and Life Science, University of Liverpool, Liverpool L3 5TR, UK; 2 Department of Plastic Surgery, Nottingham City Hospital, Nottingham NG5 1PB, UK; 3 School of Medicine, Royal College of Surgeons in Ireland, Building No. 2441, Road 2835, Busaiteen 228, Muharraq P.O. Box 15503, Bahrain; 4 Barts and The London School of Medicine and Dentistry, Queen Mary University of London, London E1 2AD, UK; 5 Northampton General Hospital, Northampton NN1 5BD, UK; asveny.rajananthanan2@nhs.net; 6 School of Medicine, Queen’s University Belfast, Belfast BT9 7BL, UK; 7 Department of Medicine, University Medical & Dental College (UMDC), Sargodha Rd, University Town, Faisalabad 38000, Punjab, Pakistan; 8 Faculty of Medicine, Ain Sham University, Cairo 11566, Egypt; 9 Department of Cardiothoracic Surgery, Liverpool Heart and Chest Hospital, Liverpool L14 3PE, UK

**Keywords:** deep sternal wound reconstruction, wound infection, multidisciplinary team, infection, flaps, surgical intervention, reconstruction, plastic surgery

## Abstract

This literature review critically examines the historical, current, and prospective dimensions of sternal wound reconstruction in the specific context of deep sternal wound infection (DSWI), aiming to enhance patient outcomes and optimise surgical techniques. Preventive measures, including prophylactic antibiotic administration and surgical site preparation, are crucial in reducing the incidence of DSWI. Effective management necessitates a multidisciplinary approach encompassing surgical debridement, drainage, and sternum repair utilising diverse procedures in conjunction with antibiotic therapy. Traditional approaches to managing DSWI involved closed irrigation and drainage techniques. While these methods exhibited certain advantages, they also exhibited limitations and varying degrees of success. The current care paradigms emphasise prophylactic antibiotic administration and surgical interventions like closed suction and irrigation, vacuum-assisted closure, and flap reconstruction. Future advancements in surgical techniques and technology hold promise for further enhancing sternal wound reconstruction. This review separates and emphasises the distinct roles of prophylaxis, antibiotic treatment, and reconstructive techniques, each relevant specifically to DSWI management. Collaborative efforts between cardiac and plastic surgeons, supported by ongoing research and innovation, are indispensable to advance sternal wound restoration and achieve superior outcomes in terms of patient welfare, morbidity and mortality reduction, and surgical efficacy.

## 1. Introduction

Deep sternal wound infections (DSWIs) are serious complications following sternotomy in cardiothoracic surgery, requiring proper reconstruction using local or locoregional flaps like pectoralis major muscle, rectus abdominis muscle, latissimus dorsi muscle, or the omentum. DSWI, characterised by deep infection within the sternum, is a significant concern in cardiac surgery, associated with prolonged hospitalisation, increased costs, and higher morbidity and mortality rates [1].

There are different types and classifications of sternal wound infections (SWIs) based on the extent and severity of infection. Superficial SWI involves infection limited to the superficial layers of the sternum, while deep SWI affects deeper tissues and structures. Complicated SWI refers to infections with additional complications like mediastinitis or osteomyelitis. The severity of the disease can range from mild local infection to severe systemic infection with a high risk of complications. The prevalence and incidence of DSWI vary based on patient factors (age and gender), surgical techniques, and preventive measures. The rates range from 0.5% to 15% among cardiac surgery patients, with higher incidences in those with additional risk factors (obesity, diabetes, smoking, advanced age, and immunosuppression) [2] and prolonged surgical duration.

Identifying risk factors is crucial for prevention. Patient-related factors and surgical factors (bilateral internal mammary artery grafts, reoperations, and prolonged cardiopulmonary bypass time) increase the risk. Inadequate infection control measures (surgical site preparation, antimicrobial prophylaxis, and wound closure techniques) contribute to SWI development. Diagnosis involves clinical assessment and imaging. Clinical signs (sternal pain, erythema, swelling, purulent discharge, fever, and elevated inflammatory markers) raise suspicion, while imaging (CT scans and MRI) confirms deep-seated infections and assesses involvement. Blood cultures and wound cultures can help identify the causative microorganisms [3]. Prompt management is essential, including surgical debridement, drainage of infected fluid collections, sternum reconstruction, and tailored antibiotic therapy guided by culture results. The choice of antibiotics should cover common pathogens associated with SWI, such as *Staphylococcus aureus*, including methicillin-resistant strains.

The severity of SWI determines the treatment approach. Mild cases may be managed with wound care, antibiotics, and close monitoring. Moderate to severe cases require surgical intervention, including debridement, irrigation, and reconstruction [4]. In cases of mediastinitis, thorough debridement and drainage of the infected mediastinum are essential.

Therefore, understanding the prevalence, risk factors, classifications, and diagnostic approaches is crucial for preventive measures, early detection, and appropriate management, improving patient outcomes in cardiac surgery.

We performed a thorough review of the literature to highlight the changes in reconstruction surgery following a DSWI as medical research advanced through the years. We also went on to analyse possible future changes in DSWI reconstruction and how they can improve treatment and results.

## 2. Past Treatment Options

Over the past few decades, various treatment methods have been developed for DSWI to improve treatment options and outcomes. These include both simple surgical techniques and more complex plastic surgery procedures. However, there is a lack of agreement on the best approach due to conflicting and inconsistent evidence regarding the superiority of specific procedures.

In 1963, continuous irrigation and drainage in a closed sternum was developed [5,6,7]. This was revolutionary, as it prevented prolonged immobilisation, intubation, and eradicated thoracic instability associated with healing. It also aided in removing and washing out large quantities of clots, fibrin, and other debris [7]. Due to its efficiency and favourable outcomes for patients, it was regarded as the standard therapy for mediastinitis globally until the 1980s.

The closed drainage and irrigation (CD-CI) technique was described using 38 patient results [8]. Firstly, all necrotic sternal bone and excess cancellous material were removed, following with repeated irrigation of the wound with copious amounts of antiseptic solution. Once the wound had been cleared of fibrinopurulent material, surgeons judged whether local damage would prevent skin closure, and if it did not, then a closed technique was applied. The sternal edges were then brought together over one or two infusion catheters and two or three large and multi-fenestrated drainage tubes. The sternum was then rewired shut, and the subcutaneous wound and skin were closed without a drain. Furthermore, postoperatively, the mediastinum was continuously irrigated with 0.5% povidone-iodine solution while simultaneously gently suctioning the wound [8]. Due to reported failure rates of up to 50%, what was once the main technique quickly diminished, since its reliability was in doubt [9,10].

In the late 1980s, Lecompte et al. proposed simple closed drainage using Redon catheters (CDRC) [11]. Redon catheter drainage, praised for its simplicity and reduced maintenance requirements when compared to closed drainage and irrigation, quickly gained support. Closed drainage using Redon catheters was achieved by placing multiple small catheters into a negatively pressurised bottle at the time of sternal debridement [8,12,13,14]. In 1989, Durandy et al. described Redon catheter use in 10 patients, with extremely successful closure rates. Durandy et al. described CD-RC as a less aggressive treatment modality compared to what was used before [11]. While this technique was less aggressive, subsequent studies, such as those by Kirsch et al. [15], emphasised the importance of aggressive debridement in cases with Methicillin-resistant *Staphylococcus aureus* (MRSA) or recurrent infections to improve patient outcomes. Furthermore, Kirsch et al. described Redon catheter primary closed drainage as the optimal therapy for many patients with poststernotomy mediastinitis. However, he cautioned that it would be more advantageous for patients with Methicillin-resistant *Staphylococcus aureus* (MRSA) or recurrent infection to proceed with a more aggressive initial debridement and procedure [15].

### 2.1. Prophylaxis and Infection Management

#### Prophylactic Antibiotics and Effective Infection Control Strategies Are Essential in Reducing Postoperative DSWI Rates

Initially, empiric antibiotic therapy provides broad coverage against various types of bacteria. Culture-directed antibiotic therapy should be started once microbiological analysis is available, while additional cultures from blood, urine, and sputum may be obtained [16]. Systemic antibiotics are typically administered for a duration of 6 weeks, with guidance from infectious disease specialists. Antifungal medications may be considered where there is no clinical improvement with antibiotics [16].

Intranasal mupirocin and systemic antibiotics have been proven to lower infection risks associated with *Staphylococcus aureus*, including methicillin-resistant strains [17]. Preoperative skin preparation and rigorous postoperative wound care protocols further enhance protection against infection [16,17].

## 3. Current Paradigms in Management

The current management approaches for deep sternal wound infections (DSWIs) involve both medical and surgical treatments [16]. 

### 3.1. Medical and Surgical Treatment

The management of DSWI relies on two key principles: firstly, the eradication of topical infection and, secondly, the stable osteosynthesis of the sternum. The eradication of topical infection can be achieved with debridement, continuous irrigation, and VAC [18].

A wide variety of surgical treatments, on the other hand, are available for sternal wound closure but will mainly focus on closed suction and irrigation, the role of VAC (vacuum-assisted closure), and flap coverage [16]. 

Closed suction and irrigation significantly advanced surgical practice [16]. This technique was improved with closed drainage, where multiple small Redon catheters are placed into a negatively pressurised bottle during sternal debridement [8,12,14,19,20], demonstrating reduced 30-day mortality and failure rates compared to other surgical methods [11].

VAC is another effective method for tissue repair following DSWI by increasing the peristernal blood flow [21,22] and facilitating wound edge approximation to offer optimal chest stabilisation [23,24]. VAC has been promising, particularly for critically ill patients who cannot tolerate extensive reconstructive surgery [24].

Although these methods are effective, they often fail to cure the patient [18]. DSWI is a grave complication, imposing significant morbidity and mortality risks. The incidence rates of DSWI are influenced by surgical variables and patient characteristics. Therefore, identifying risk factors and utilising clinical evaluation, along with diagnostic imaging, are essential preventive measures.

However, should patients develop DSWI and are a good surgical candidate, upon eradication of the infection, osteosynthesis of the sternum should be achieved with sternal fixation and flap coverage [18].

### 3.2. Sternal Fixation

Sternal fixation can be achieved with the use of episternal devices such as rigid plate fixation or wire cerclage. Wire cerclage involves the use of stainless steel or titanium wires, which are threaded through predrilled holes in the sternum and twisted to provide stabilization. Rigid plate fixation is also another episternal device, which involves titanium or stainless steel plates secured to sternum superiorly using screws, enabling rigid fixation of the sternum [19,20]. In managing DSWI, surgical techniques like cerclage wiring and episternal fixation with titanium plates remain critical in maintaining sternal stability. Cerclage wiring is widely used due to its simplicity, cost-effectiveness, and efficacy in most DSWI cases without severe sternal instability [4,16]. For high-risk patients or those with prior sternotomies, episternal fixation using rigid titanium plates offers reduced sternal complications [19].

### 3.3. Flap Reconstruction

Flap reconstruction, considered as the standard therapy for DSWIs, offers early wound closure and reduces mortality [25,26,27]. Research has demonstrated a review of 211 sternal infections that are treated with pedicled muscle flaps with a successful wound closure rate of 95% alongside a mortality rate of 5.7% [28]. The omental flap is another useful reconstructive option due to its ability to conform to the deepest recesses of the sternal wound and carries immunologic properties, with data showing superiority over the pectoralis flap in preventing sepsis-related morbidities [28]. In contrast, the rectus abdominus flap is supported due to its convenient dissection process and its ability to rotate widely. This enables the flap to reach not only the sternal notch but also the lower third of the sternal wound, which is frequently prone to complications [16].

The current major methods for DSWI reconstruction depend on whether they are adequate for pectoralis muscle and soft tissues [29]. Both methods discussed will only be plausible under the assumption of adequate debridement and cultures [29]. Pectoralis flaps will be the method of choice when there is sufficient muscle and soft tissues present; otherwise, the omental or rectus flap will be the method of choice [29].

**Figure 1:**
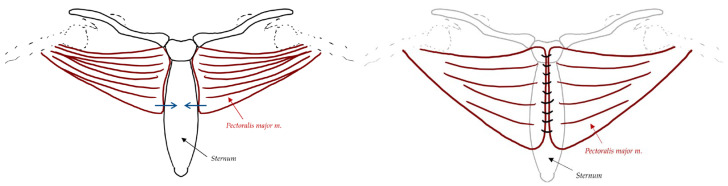
Pectoralis major myocutaneous advancement flaps. Flaps are elevated posterior to the muscle, where it is relatively avascular. Dissection occurs medially to laterally and ends when the muscle reaches the midline with minimal tension. Both flaps are sutured together in the midline with the pectoralis fascia and rectus sheath.

Pectoralis major myocutaneous flaps are elevated in the avascular plane just beneath the pectoralis major muscle from the central to the lateral direction [29]. The dissection of the flap is ceased as soon as the flaps can be advanced to the centre with minimal tension, which usually involves dissecting to the area between the midclavicular and anterior axillary lines, whereas the dissection stops just inferior to the clavicle superiorly and deep to the anterior rectus sheath to the level of the xiphoid process inferiorly (Figure 1) [29]. Closed suction drains will be placed beside each flap, with a third one placed centrally over the mediastinum [29]. Flaps will be sutured together with interrupted no. 2 Vicryl or polysorb sutures, with the pectoralis fascia and the rectus sheath in the same closure layer [29]. The only recognised downside to the pectoralis flap is the limited coverage over the xiphoid and inferior portion of the wound due to the limited extension of the pectoralis major muscle [29]. This, however, can be overcome by bringing the anterior rectus sheath in continuity with the pectoralis major flap [29].

**Figure 2:**
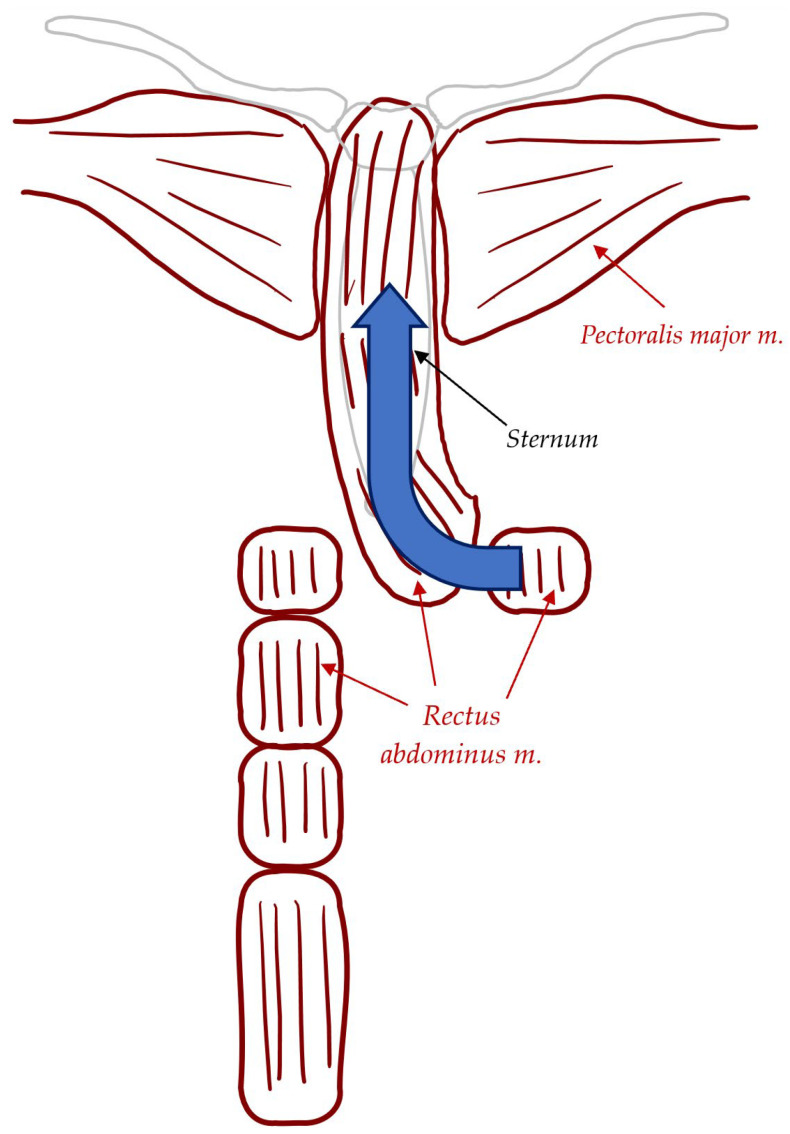
Rectus abdominus flap. This pedicled flap is derived from the rectus abdominis muscle, with its blood supply originating from the superior epigastric artery. The muscle is divided at its inferior aspect and rotated superiorly to cover the sternal defect.

In cases where the pectoralis major muscles are compromised and a patent ipsilateral IMA (internal mammary artery), rectus abdominis muscle can be harvested in the reconstruction of sternal wound defects [29]. As a pedicled muscle flap based on the SEA (superior epigastric artery), it can be divided at the inferior most portion and rotated superiorly. Figure 2 demonstrates this. In cases where the pectoralis major muscles are compromised and a patent ipsilateral internal mammary artery (IMA) is present, rectus abdominis muscle can be employed in the reconstruction of sternal wound defects [29]. Rectus abdominis muscle flaps are able to provide more stable coverage of inferior sternal wound defects; however, more complications such as abdominal wall weakness, bulge, or hernia may arise due to the requirement of a second donor site for the flap [29].

**Figure 3:**
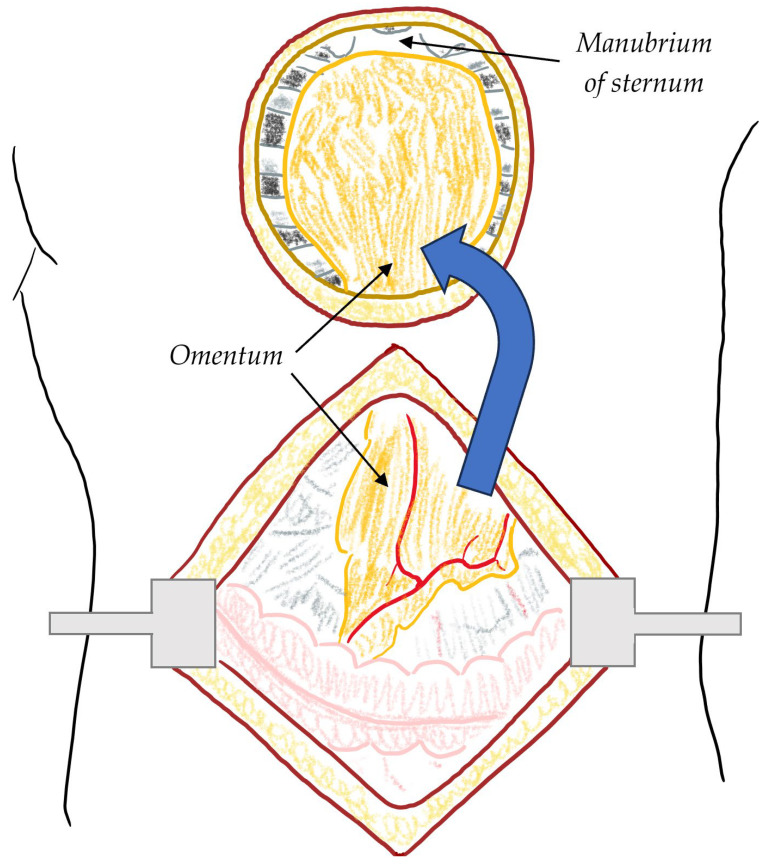
Omental flap. The omentum is harvested from the abdomen, carefully preserving its attachment to the gastroepiploic arteries. It is then transposed to the chest through an opening in the diaphragm or abdominal fascia, allowing it to reach the sternum, where it is utilised to cover and fill the sternal defect.

On the other hand, the omentum flap remains a strong secondary option for patients with extensive loss of chest wall soft tissue and inadequate skin for closure, especially for lower third infections [29]. Figure 3 shows the process of omental flap formation. It remains the second-line intervention due to its difficult harvesting nature (via laparotomy), less tissue bulk provided, and no extra support to chest wall stability [29]. Other considerations for the omentum flap is the necessary creation of an abdominal fascia or diaphragm opening in order for the omentum flap to reach the sternum, with the possible need for skin grafting [29]. Table 1 describes the reconstruction methods for DSWI as above.

#### 3.3.1. Advantages and Disadvantages of Sternal Wound Reconstruction

One of the main advantages of plastic surgery reconstruction for DSWI is improved wound healing. Plastic surgeons utilise muscle flaps, which bring well-vascularised tissue to the wound site, enhancing blood supply and reducing the risk of infection [5]. This approach promotes faster healing, decreases wound complications, and improves overall outcomes. Furthermore, plastic surgeons prioritise aesthetic outcomes and employ meticulous techniques to minimise scarring, resulting in a more cosmetically appealing result. They also focus on recreating a natural sternal appearance using tissue flaps, enhancing patient satisfaction with their postoperative appearance [30]. Additionally, plastic surgeons aim to restore full functional integrity by employing techniques that provide stability and protection to the underlying structures, reducing the risk of complications and improving the patient’s functional outcome [31].

However, there are disadvantages to sternal wound reconstruction from a plastic surgery perspective. Performing these surgeries requires complexity and expertise. Plastic surgeons must have a thorough understanding of the underlying anatomy, wound healing principles, and reconstructive techniques specific to sternal wounds. This specialised knowledge may limit the availability of experienced plastic surgeons who can perform these procedures. Moreover, sternal wound reconstruction surgeries can be prolonged and time-consuming [32]. They involve meticulous dissection, tissue transfer, and suturing, resulting in extended operation times compared to simple wound closure surgeries. Prolonged surgery increases the risk of intraoperative complications and may require more extensive anaesthesia, which carries associated risks [33].

The most common risks and complications of complex and prolonged sternal wound reconstruction surgeries include flap necrosis, infection, hematoma, seroma formation, and wound dehiscence. These complications can extend the wound healing time, necessitate additional surgical interventions, and potentially impact the patient’s recovery and overall outcome [34]. Due to the specialised care and knowledge required, there may be a limited availability of plastic surgeons specialising in sternal wound reconstruction, which can result in delays in treatment and limit the options available to patients requiring this procedure [35].

Overall, it is important to consider advantages and disadvantages according to each patient’s individual factors, such as the extent of the wounds, age, gender, mortalities, etc. Consulting a specialist plastic surgeon is essential to assess the feasibility, risks, and potential benefits of the procedure in each case [36].

Table 2 summarises the advantages and disadvantages of utilising plastic surgery for deep sternal wound reconstruction.

#### 3.3.2. Comparison on Types of Flaps/Reconstruction Techniques

DSWI requires reconstructing sternal wounds using various techniques. Factors such as infection severity, patient comorbidities, and surgeon expertise influence the choice of technique [37]. Treatment involves drainage, debridement, and antibiotics, followed by sternum and tissue reconstruction for organ protection and chest wall stability [37]. The literature offers insights on different flap options and comparative analyses for sternal reconstruction, aiding in selecting appropriate techniques [37].

The combination of negative pressure therapy and pectoralis major muscle flap coverage has proven to be an effective treatment for deep sternal wound infections after cardiothoracic surgery [38]. A study involving 167 patients showed that monolateral flap reconstruction resulted in fewer complications, a shorter hospital stay, and a statistically significant difference in the hematoma rate [38]. The chimeric anterolateral thigh perforator flap has demonstrated effectiveness in controlling infection and eliminating deep dead space in complex limb wounds, with potential for broader clinical application [39]. On the other hand, a review of 16 studies comprising 1046 patients suggests that omental flaps may be associated with lower mortality and fewer complications compared to muscle flaps [40]. A meta-analysis of four studies involving 528 patients found no significant differences in postoperative outcomes between the pectoralis major flap and omental flap for sternal reconstruction [41].

Utilising omental flaps and bipectoral musculofascial advancement flaps has been deemed an effective strategy for reconstructing infected sternal wounds in the inferior areas, providing a comprehensive surgical solution [40]. Various flap types can be utilised for sternal reconstruction. Pectoralis major flaps are effective and less invasive than muscle flaps, but they may result in long-term functional impairment [42]. Rectus abdominis flaps are superior to pectoral flaps in terms of coverage of the inferior sternum and can maintain a viable flap even with ipsilateral internal mammary artery (IMA) ligation [43]. Omental grafts are beneficial for filling dead spaces and possess large pedicles, rich vascular networks, and lymphatic networks, although they may be susceptible to secondary cancers [44]. Latissimus dorsi flaps involve the intrathoracic transposition of extrathoracic skeletal muscle and are useful for intrathoracic infections associated with airway, lung parenchyma, oesophageal, cardiac, or great vessel complications [45]. Tensor fascia lata flaps are suitable for covering the upper third of the sternum and have well-established vascular anatomy [46]. Free flaps and perforator flaps can be employed for significant defects, but they require longer operating times, carry a risk of flap failure, demand microsurgical expertise, and result in higher costs [47]. The vastus lateralis myocutaneous flap has a high success rate, while the rectus abdominis and latissimus dorsi/parascapular flaps have lower success rates [48]. Internal mammary artery perforator flaps have shown promise in sternal reconstruction, but further research is necessary to confirm their efficacy [49].

Table 3 summarises the various flaps and reconstruction techniques discussed for sternal wound complications.

## 4. Discussion

### 4.1. Future Direction for Mediastinitis Treatment Using Sternal Wound Reconstruction

Managing deep sternal wound infection (DSWI) remains challenging, with mediastinitis being the most severe and life-threatening complication. Sternal wound reconstruction (SWR) is a crucial component of DSWI management, aimed at stabilising the sternum, restoring the chest wall contour, and promoting wound healing. Unfortunately, despite significant advances in SWR techniques, mediastinitis remains associated with high morbidity and mortality rates. While plastic surgery techniques, such as muscle flaps, are critical for severe cases of DSWI, simpler and more widely used methods, including cerclage wiring and titanium plate fixation, remain effective first-line treatments for most cases of DSWI. This section will explore the future directions for mediastinitis treatment using SWR, focusing on recent developments and trends in the field.

Future advancements in DSWI treatment will likely continue to rely on both plastic and cardiac surgery techniques. While flap reconstruction remains a vital option for cases with extensive tissue loss, advancements in sternum fixation devices, such as 3D-printed titanium plates, are expected to further improve outcomes in complex cases [58,59,60,61]. Innovations in cerclage wiring and episternal devices will likely make these cardiac techniques even more applicable in treating a wider range of DSWI patients.

### 4.2. Surgical Techniques

Surgical techniques for SWR have evolved significantly in recent years. Yu et al. evaluated the outcomes of various SWR techniques, including pectoralis muscle flaps, omental flaps, and titanium plates [61]. The study found that pectoralis muscle flaps had the highest success rates, while titanium plates had the lowest success rates [61]. However, titanium plates were associated with lower morbidity rates and shorter hospital stays than the other techniques. A recent study by Lei Sun et al. also demonstrated the feasibility and efficacy of a novel SWR technique involving a three-dimensional-printed titanium implant [62].

#### 4.2.1. Allogenic Flaps

Allogenic flaps have emerged as a promising alternative for SWR in cases of complex DSWI with extensive tissue loss [63]. These flaps are derived from cadaveric or living donors, processed and stored in tissue banks, and later implanted in the recipient [63]. Recent studies have demonstrated the safety and effectiveness of allogenic flaps for SWR, with low rates of flap-related complications and good long-term outcomes [63].

#### 4.2.2. Breast Flaps

Breast flaps represent another alternative for SWR, particularly in female patients who have undergone mastectomy. These flaps are based on the pectoralis major muscle and overlying skin, which are harvested from the contralateral breast or the same breast in cases of bilateral mastectomy. Recent studies have reported good outcomes with breast flaps for SWR, with low rates of flap-related complications and good aesthetic results. Matsen et al. found that, out of 606 consecutive procedures, flap necrosis, infection, and wound dehiscence were low at 14% overall [64]. The review concluded that breast flaps are a feasible and effective option for SWR in female patients with DSWI who have undergone mastectomy [64].

While plastic surgery techniques such as flap reconstruction are critical in severe DSWI cases, traditional cardiac surgery approaches like cerclage wiring and sternum plating remain the primary options in many cases. Cerclage wiring, due to its simplicity and cost-effectiveness, is frequently used in primary closure cases, especially when sternal instability is not severe. Additionally, episternal fixating devices, particularly titanium plates, have shown superior mechanical stability in complex or high-risk patients. These techniques are essential in achieving proper sternal fixation, promoting stability, and reducing the risk of long-term complications.

#### 4.2.3. Cerclage Wiring

Cerclage wiring is one of the most commonly used techniques for sternal reconstruction, particularly in cases of primary closure after cardiothoracic surgery. It involves using stainless steel wires to stabilise the sternum. Cerclage wiring is widely regarded for its simplicity, cost-effectiveness, and accessibility. It is particularly useful in low-risk patients without complicating factors like severe infection or sternal instability. However, in high-risk patients, such as those with prior sternotomy or osteoporosis, cerclage wiring may result in inadequate stabilisation, leading to sternal instability or infection. Studies have shown varying success rates, depending on the patient’s overall health condition and surgical history. Despite its limitations, cerclage remains a routine practice due to its simplicity and low cost.

#### 4.2.4. Episternal Fixating Devices

Episternal fixating devices, including rigid titanium or polymer plates, are commonly used in more complex sternal reconstruction cases, particularly when sternal instability or sternal dehiscence is present. These devices provide superior mechanical stability compared to cerclage wiring, reducing the risk of postoperative sternal dehiscence or instability. Plates can be particularly useful in patients with complicating factors like obesity or a history of sternotomy, where additional support is required. However, the main disadvantages include higher cost, more complicated surgical procedures, and the need for specialised equipment and expertise. Despite these limitations, many studies have reported a significant reduction in postoperative morbidity and mortality in patients treated with plates versus traditional cerclage wiring.

The current techniques, such as cerclage wiring and episternal fixating devices, remain widely adopted in sternal reconstruction. Cerclage wiring offers a straightforward, low-cost solution for primary closure but may present limitations in more complex cases, particularly with high-risk patients, where sternal instability can occur. On the other hand, episternal fixating devices, such as titanium plates, offer superior sternal stability and have been associated with reduced morbidity and mortality, especially in high-risk populations. However, they require advanced equipment and are significantly more expensive. Emerging techniques, such as allografts and gene therapy, hold promise for future applications but are still in the experimental stages and lack the robust clinical data available for the more traditional methods. Table 4 summarises the advantages and disadvantages of each technique.

### 4.3. Biomaterials for Sternal Wound Reconstruction

Recent studies have highlighted the importance of biomaterials in sternal wound reconstruction. Researchers have been investigating new materials that can promote tissue regeneration and reduce the risk of infection. Mohammadyari et al. (2023) found that using an acellular dermal matrix (ADM) as a scaffold for sternal wound reconstruction resulted in lower infection rates and higher wound healing rates than the traditional method [65]. Additionally, a review by Sharma et al. (2022) showed that using collagen-based biomaterials in sternal wound reconstruction resulted in improved outcomes compared to traditional methods [66]. Furthermore, a recent study by Guo et al. (2022) demonstrated the feasibility and efficacy of using adipose-derived stem cells (ASCs) for SWR [67]. The study found that ASCs were associated with improved wound healing and reduced scarring [67].

### 4.4. Minimally Invasive Techniques for Sternal Wound Reconstruction

Traditional vacuum-assisted closure (VAC) therapy has remained a key treatment modality for managing deep sternal wound infections (DSWIs), particularly for patients too critically ill to undergo immediate reconstructive surgery. A study by Hämäläinen et al. (2021) compared the use of VAC therapy to early reconstructive treatments and found that, while both approaches were effective, VAC therapy provided significant benefits in reducing infection rates and facilitating wound healing in the early stages of treatment [68]. Rather than representing a novel or minimally invasive approach, VAC therapy remains a well-established method that continues to play a vital role in DSWI management.

### 4.5. Antibiotic-Loaded Biomaterials for Infection Prevention

Infection prevention is a crucial aspect of sternal wound reconstruction. Antibiotic-loaded biomaterials have been investigated to prevent infection and promote wound healing. A systematic review by Jiang et al. (2022) found that antibiotic-loaded bone cement effectively reduced the risk of infection in sternal wound reconstruction procedures [18]. Additionally, a study by Martino et al. (2020) showed that using antibiotic-loaded ADM resulted in lower infection rates and improved wound healing compared to traditional methods [69].

### 4.6. Gene Therapy for Tissue Regeneration

Gene therapy has emerged as a promising approach for tissue regeneration and repair. Researchers have been investigating the use of gene therapy in sternal wound reconstruction to promote tissue regeneration and reduce the risk of infection. A study by Zang et al. (2022) showed that using adenovirus-mediated delivery of bone morphogenetic protein-2 (BMP-2) in sternal wound reconstruction improved bone regeneration and reduced infection rates [70]. Table 5 summarises the future techniques for mediastinitis treatment using sternal wound reconstruction.

### 4.7. Psychological Impact on Patients

DWSI is a morbid and costly complication. Data describing patients’ long-term outcomes, quality of life, and psychological impact after deep sternal wound infections are scarce. A sternal wound infection often leaves patients with physical, cosmetic, and mental scars associated with prolonged hospital stays and an estimated mortality of 10% while leading to a decreased quality of life [71,72]. Furthermore, wound infections have been proven to have a vital impact on surgical costs, repeated procedures, and a possibly lifelong psychological consequence [73,74]. Employing plastic surgery reconstruction following DWSI has many advantages for the patients, including improved wound healing and, consequently, decreased wound complications. Measuring quality of life is an important aspect of delivering a holistic surgical approach. The presence of scars can result in mental disturbances in patients with associated symptoms of anger, anxiety, depression, and post-traumatic stress [74]. Consequently, plastic surgeons will prioritise satisfactory aesthetic outcomes in sternal wound reconstruction when compared to other surgeons such as cardiac surgeons. They will achieve this through minimally invasive scarring techniques that will positively impact patients’ psychological well-being moving forward.

**Figure 4:**
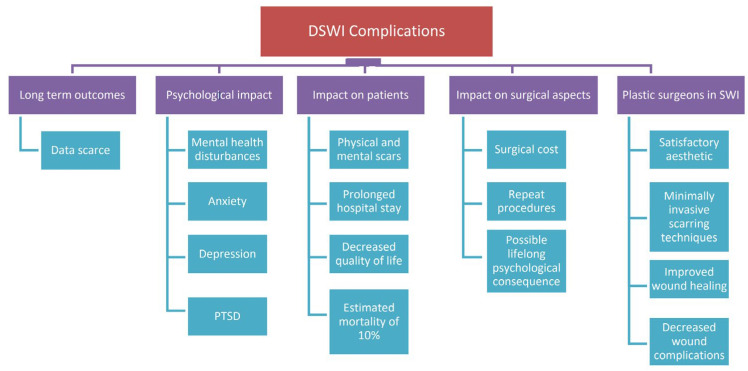
Illustrates the impact of deep sternal wound infections (DSWIs) on patients’ long-term outcomes, quality of life, and psychological well-being.

Figure 4 illustrates the impact of deep sternal wound infections (DSWIs) on patients’ long-term outcomes, quality of life, and psychological well-being.

## 5. Conclusions

DSWI is a serious complication after cardiothoracic surgery, and its occurrence varies based on patient and surgical factors. Identifying risk factors and preventive measures are crucial. Prompt diagnosis through assessment and imaging is essential. Timely management, including debridement, drainage, and reconstruction, is important for preventing complications. However, there is no consensus on the optimal treatment approach, requiring further research. Plastic surgery offers advantages like improved wound healing, aesthetics, and functional integrity. However, it requires specialised expertise and entails risks and complications. Individual patient factors should guide the choice of sternal wound reconstruction approach.

## Figures and Tables

**Table 1:** Reconstruction methods for DSWI.

Reconstruction Methods for DSWI	Description
Pectoralis Major Myocutaneous Flaps	Flaps elevated beneath the pectoralis major muscle from central to lateral direction. Dissection stops between midclavicular and anterior axillary lines, inferior to the clavicle superiorly, and deep to anterior rectus sheath to xiphoid process inferiorly. Closed suction drains placed beside each flap. Flaps sutured together with interrupted sutures (Vicryl or polysorb) with pectoralis fascia and rectus sheath in the same closure layer, Limited coverage over xiphoid and inferior portion of the wound, which can be overcome by bringing anterior rectus sheath in continuity with the pectoralis major flap
Rectus Abdominis Muscle Flaps	Harvested when pectoralis major muscles are compromised, and a patent ipsilateral IMA is present. Pedicled flap based on the superior epigastric artery (SEA). Divided at the inferior most portion and rotated superiorly. Provides more stable coverage but may lead to complications like abdominal wall weakness, bulge, or hernia due to requiring a second donor site for the flap.
Omentum Flap	Secondary option for extensive loss of chest wall soft tissue and inadequate skin for closure, particularly for lower third infections. Harvesting via laparotomy. Provides less tissue bulk and no extra support to chest wall stability. May require creation of abdominal fascia or diaphragm opening for the omentum flap to reach the sternum, possibly needing skin grafting.
Cerclage Wiring	Stainless steel wires used to stabilise the sternum in primary closure, particularly in low-risk patients without sternal instability. Simple and cost-effective.
Episternal Fixating Devices (Plates)	Titanium plates providing mechanical stability in cases of sternal instability or complex reconstructions. Superior fixation for high-risk patients or those with prior sternotomies.

**Table 2:** Advantages and disadvantages of sternal wound reconstruction from a plastic surgery perspective.

Advantages	Disadvantages
Improved wound healing	Complexity and expertise required
Promotes better wound healing	Limited availability of experienced plastic surgeons
Reduced risk of infection	Prolonged and time-consuming surgery
Faster healing and decreased complications	Increased risk of intraoperative complications
Improved aesthetic outcomes	Risk of flap necrosis, infection, hematoma, seroma formation, and wound dehiscence
Enhanced patient satisfaction with appearance	Limited availability of specialised plastic surgeons
Restoration of functional integrity	Delays in treatment due to limited options

**Table 3:** Comprehensive comparison of flap reconstruction techniques.

Flap/Reconstruction Technique	Advantages	Disadvantages	Reported Success Rates
**Pectoralis Major Flaps**	-Provides a good vascular supply unaffected by internal mammary artery harvesting and is relatively simple to harvest [29]. -Offers coverage of exposed vital structures [29]. -Provides ribcage stability without the need for osteosynthesis [50,51]. -Preserves strength in at least one arm, with the possibility of preserving the contralateral muscle group in case of surgical failure [52]. -Demonstrates resistance to wound infection [53,54].	-May cause long-term functional impairment. -Loss of skeletal continuity of the chest wall may be more disabling than the loss of pectoral muscle function.	A study from Fujian Medical University involving 11 patients undergoing sternal wound reconstruction with this method reported no bleeding or secondary thoracotomies, with drains removed within 7 days and sutures removed within 14 days post-op [42]. Only one patient experienced secondary healing, with no subcutaneous hematoma or bleeding requiring further surgery. Six-month follow-up showed no postoperative complications, including pain, abnormal upper limb movement, or chest wall deformities [42]. Another study at Linköping University Hospital reviewed outcomes of 43 flaps; 37 were successful, with 3 failed procedures associated with higher BMI (31.1 versus 27.8), older age (78.6 versus 74.4), and more pre-existing conditions [52]. Postoperative complications were recorded in 49% of patients, including bleeding, infection, wound dehiscence, fistulation, and skin necrosis [52].
**Rectus Abdominis Flaps**	-Superior to pectoralis major flaps for covering the inferior sternum [55]. -Can maintain a viable flap even with ipsilateral IMA ligation [43]. -Capable of transferring large skin areas with varying thickness and amounts of underlying muscle [50].	-May require arterial and/or venous re-charging. -May be less effective than pectoral flaps in certain cases [56]. -Associated with an increased risk of surgical site infection (SSI). In one study, all 5 patients undergoing VRAM (vertical rectus abdominis myocutaneous) flap reconstructions experienced postoperative complications [50].	A case report involving 5 male chronic smokers (age range: 42–74, mean age: 58) demonstrated 100% muscle survival. All patients experienced good healing and infection-free wounds [43].
**Omental Grafts**	-Large size and bulk help fill dead spaces. -Rich vascular and lymphatic networks, with large pedicles, make them particularly useful in managing infection-related sternal wound complications.	-May be prone to malignancies originating from a primary cancer. -Associated with higher rates of reoperation (18%), SSI (17%), skin necrosis (4.7%), flap necrosis (3.8%), dehiscence (3.8%), hematoma (2.8%), and mortality (2%) [50].	In a study of laparoscopic omental harvests, 7 out of 9 procedures were used for reconstruction of infection-related sternal wounds, and 2 for the repair of intrathoracic viscera. The study showed excellent early outcomes, with no late flap failures, an 8.3% mortality rate, and a mean hospital stay of 59 days for patients with DSWIs who underwent omental transposition [56,57].

**Table 4:** The pros and cons of future directions for mediastinitis treatment using SWR.

Technique	Pros	Cons
**Plates (Titanium)**	Stable fixation, reduced recovery time	Expensive, requires specialised tools
**Cerclage Wiring**	Simple, cost-effective, widely used	Risk of sternal instability, infection
**VAC Therapy**	Promotes blood flow, reduces infection	Requires specialised equipment
**Muscle Flaps**	Effective in high-risk patients	Complex surgery, cosmetic concerns
**Allografts (Emerging)**	Promising tissue regeneration	Experimental, lack of long-term data
**Gene Therapies (Emerging)**	Potential for revolutionary healing	Expensive, not yet widely available

**Table 5:** Future directions for mediastinitis treatment using sternal wound reconstruction.

Future Directions for Mediastinitis Treatment Using Sternal Wound Reconstruction	Examples
Surgical Techniques	Pectoralis muscle flaps, omental flaps, titanium plates, three-dimensional-printed titanium implant, allogenic flaps, breast flaps
Biomaterials for Sternal Wound Reconstruction	Acellular dermal matrix (ADM), collagen based biomaterials, adipose-derived stem cells (ASCs)
Minimally Invasive Techniques for Sternal Wound Reconstruction	Closed-chest vacuum-assisted closure (VAC) system
Antibiotic-loaded Biomaterials for Infection Prevention	Antibiotic-loaded bone cement, antibiotic loaded ADM
Gene Therapy for Tissue Regeneration	Adenovirus-mediated delivery of bone morphogenetic protein-2 (BMP-2)

## Data Availability

All data are found within this publication.
